# Supplementary material for: Sputum Microbiome and Chronic Obstructive Pulmonary Disease in a Rural Ugandan Cohort of Well-Controlled HIV Infection
Source: Microbiol Spectr. 2023 Feb 15;11(2):e02139-21. doi: 10.1128/spectrum.02139-21 (PMC10100697; doi:10.1128/spectrum.02139-21)
Supplement: Supplemental file 1 — Supplemental material. Download spectrum.02139-21-s0001.pdf, PDF file, 1.7 MB [file spectrum.02139-21-s0001.pdf]

Sputum microbiome and Chronic Obstructive Pulmonary Disease in a rural Ugandan cohort of well-controlled HIV

Alex Kayongo*[1-2], Theda Ulrike Patricia Bartolomaeus*[3,4,5,6], Till Birkner*[4,5,6], Lajos Markó[4,5,6,7], Ulrike Löber[4,5,6,7], Edgar Kigozi[2], Carolyne Atugonza[2], Richard Munana[10], Denis Mawanda[1], Rogers Sekibira[1], Esther Uwimaana[1,2], Patricia Alupo[1], Robert Kalyesubula[8,9], Felix Knauf[10], Trishul Siddharthan[11], Bernard S Bagaya[2], David P Kateete[2], Moses L Joloba[2], Nelson K Sewankambo[9], Daudi Jjingo[12,13], Bruce Kirenga[1,9], William Checkley[14,15] and Sofia K. Forslund[4,5,6,7,16].

Supplementary Figure 1 (Figure S1): Raw reads from 16S sequencing of induced sputum samples from the rural Ugandan cohort stratified by HIV and COPD status. Box plots represent the mean and 95% confidence interval for samples stratified into COPD-/HIV-, COPD+/HIV-, COPD-/HIV+, and COPD+/HIV+ groups (* indicate statistically significant results, pairwise Wilcox test corrected for multiple testing using FDR).

Supplementary Figure 2 (Figure S2): Differential effect of disease status on individual microbiome features. Volcano plot showing correlation of bacterial genera with the disease status represented by effect sizes (Spearman's Rho, Cliff's delta). Yellow dots show a uniquely significant (FDR-adjusted nested mixed model LRT $Q < 0.1$) correlation with HIV status. Brown dots show a uniquely significant (FDR-adjusted nested mixed model LRT $Q < 0.1$) correlation with COPD status. Red dots show a significant (FDR-adjusted nested mixed model LRT $Q < 0.1$) correlation with both HIV and COPD status. Black circles indicate features showing significant association with the COPD+/HIV+ group ('dual disease status'), indicating additive disease signature for HIV and COPD in these features. Green dots represent features tested, showing no correlation with either of the disease statuses.

Supplementary Figure 3 (Figure S3): Effect of airway microbial diversity on lung function. Scatter plot showing the relationship between lung function scores: pre- and post-bronchodilator FVC, FEV_1 and FEV_1/FVC ratio and alpha diversity. Linear regression analysis was performed between lung function scores and alpha diversity scores (Shannon index). The shaded regions are 95% confidence intervals for the slope of the line. No statistically significant findings (Pearson correlation, $p > 0.05$).

Supplementary Figure 4 (Figure S4): Differential effect of disease status on individual GMM and KEGG module features. Impact of Disease Status, Medication, and other collected Metadata variables on the functional profile of the sputum microbiota. Heatmap shows KEGG modules significantly [MWU (for categorical factors) and Spearman (for continuous features)

*FDR<0.1, **FDR<0.01, ***FDR<0.001] different in abundance (KEGG modules) depending on Disease Status (HIV/COPD) alongside participant characteristics. Heatmap cells show effect size (Cliff's Delta for categorical factors, Spearman's Rho for continuous features). Multi-confounder testing (nested linear model testing, post hoc test) was applied, showing no stars or circles if not significant (NS) in the naive test step. In the remaining naive-significant associations, only those passing the deconfounding step as strictly deconfounded (SD), laxly deconfounded (LD) or no other covariates (NC) are black stars. At the same time, any confounded signal is grey circle.

Supplementary Figure 5 (Figure S5): Microbial community characterization in all sputum samples for both United Kingdom and Uganda samples combined. A) Clustering (community typing) of all samples based on a Dirichlet-multinomial model. The model was fitted on the genus relative abundance count matrix to classify genus abundance based on probability. The best fit for the tested data shows four Dirichlet multinomial groups (k), further named community types 1, 2, 3 and 4. Top drivers of community type 1: a mixture of *Streptococcus*, *Bacillus*, *Rothia;* community type 2: predominantly *Streptococcus*; community type 3: predominantly *Neisseria* and *Streptococcus* and community type 4: a mixture of *Streptococcus, Prevotella, Fusobacterium, Haemophilus*, and *Neisseria*. **B)** Principal coordinates analysis (PCoA) plots of Bray–Curtis dissimilarity of samples coloured according to their predicted community type (significance based on PERMANOVA). Marginal density plots depict sample group distribution alongside PCo1 and PCo2, respectively. The ellipses represent a 95% confidence interval for each cluster. **C)** Community Type distribution (vertical axis) stratified for samples origin from Uganda and UK . reported significance (***: q < 0.0001) based on pairwise chi-square-testing (FDR correction for multiple testing).

Supplementary Figure 6 (Figure S6): Impact of the cohort (UK vs Uganda) and clustered community type on the taxonomic composition of the sputum microbiome. **A).** Principal coordinates analysis (PCoA) plots of Bray–Curtis dissimilarity of samples coloured according to their predicted community type, geographical origin (n = 200 from Uganda and n = 102 from the UK), and HIV status. Significance based on PERMANOVA. Marginal density plots depict sample group distribution alongside PCo1 and PCo2, respectively. The ellipses represent the 95% confidence interval for each cluster. **B)** Alpha diversity (Shannon index) of the sputum microbiota stratified for geolocation. Significant differences between the two cohorts (Uganda and the UK) (pairwise Wilcox test corrected for multiple testing using FDR). **C)** Heatmap shows all phylum-level taxa significantly [MWU (for categorical factors) and Spearman (for continuous features)

*FDR<0.1, **FDR<0.01, ***FDR<0.001] different in abundance (binned rarefied 16S gene counts) depending on cohort alongside HIV status. Heatmap cells show effect size (Cliff's Delta for categorical factors, Spearman's Rho for continuous features). Multi-confounder testing (nested linear model testing, post hoc test) was applied, showing no stars or circles if not significant (NS) in the naive test step. In the remaining naive-significant associations, only those passing the deconfounding step as strictly deconfounded (SD), laxly deconfounded (LD), or no other covariates (NC) are black stars. At the same time, any confounded signal is grey circle.

Supplementary Figure 7 (Figure S7) : Overview schematic of metadeconfoundR statistical methods: (left) naive association testing of individual features and covariates using rank-based tests. (middle) nested model post-hoc linear model likelihood ratio tests to determine relative redundancy between covariates associated with the same feature. (right) label assignment based on likelihood ratio test outcome for each feature, covariate combination based on initial naive tests, and linear model testing results.

Supplementary Table 1 (Table ST1): Raw read count before and after filtering for potential human contamination for each sample

Figure S1

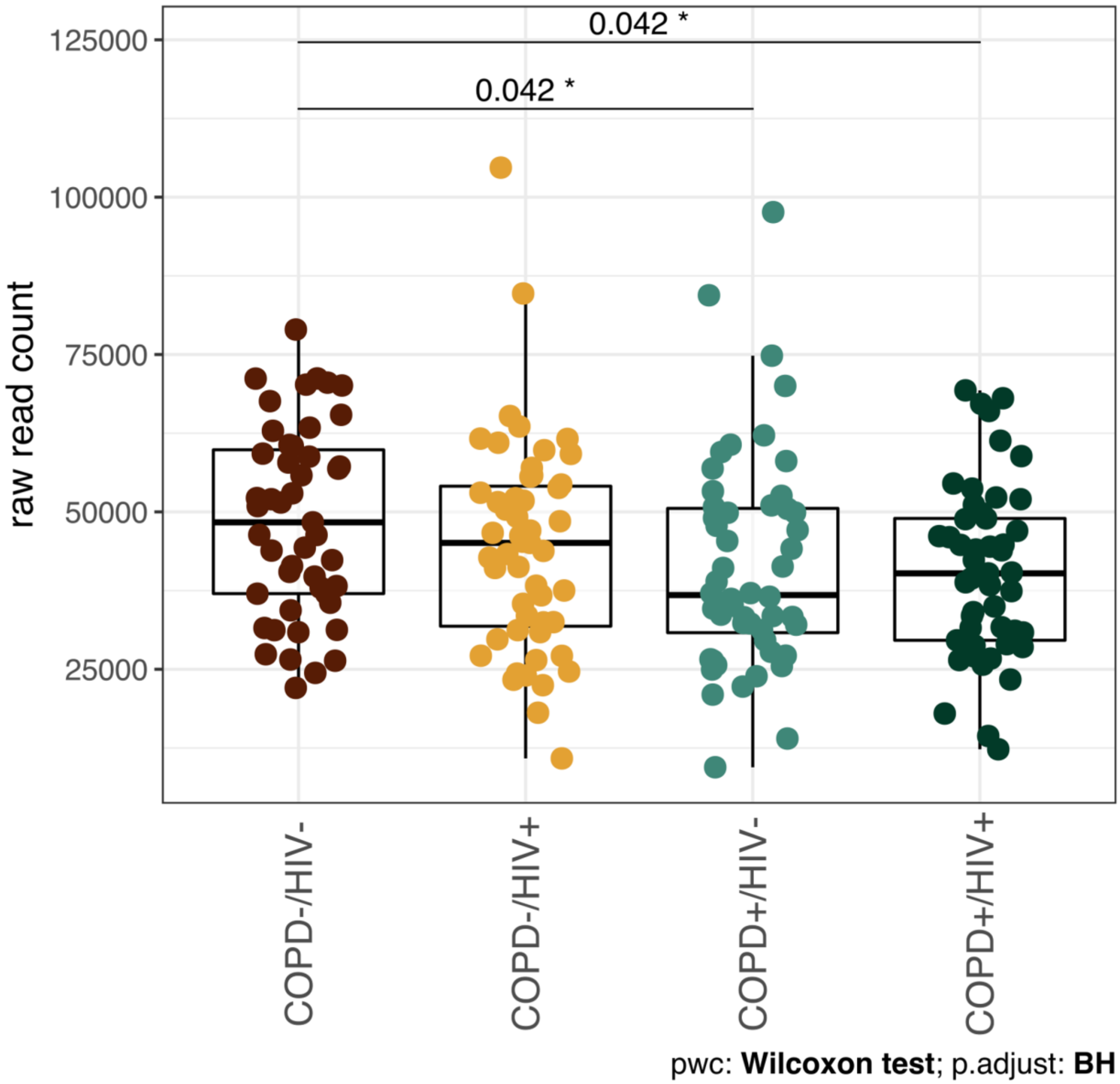
0.042 *
0.042 *
125000
100000
75000
50000
25000
raw read count
COPD-/HIV-
COPD-/HIV+
COPD+/HIV-
COPD+/HIV+
pwc: **Wilcoxon test**; p.adjust: **BH**

Figure S2

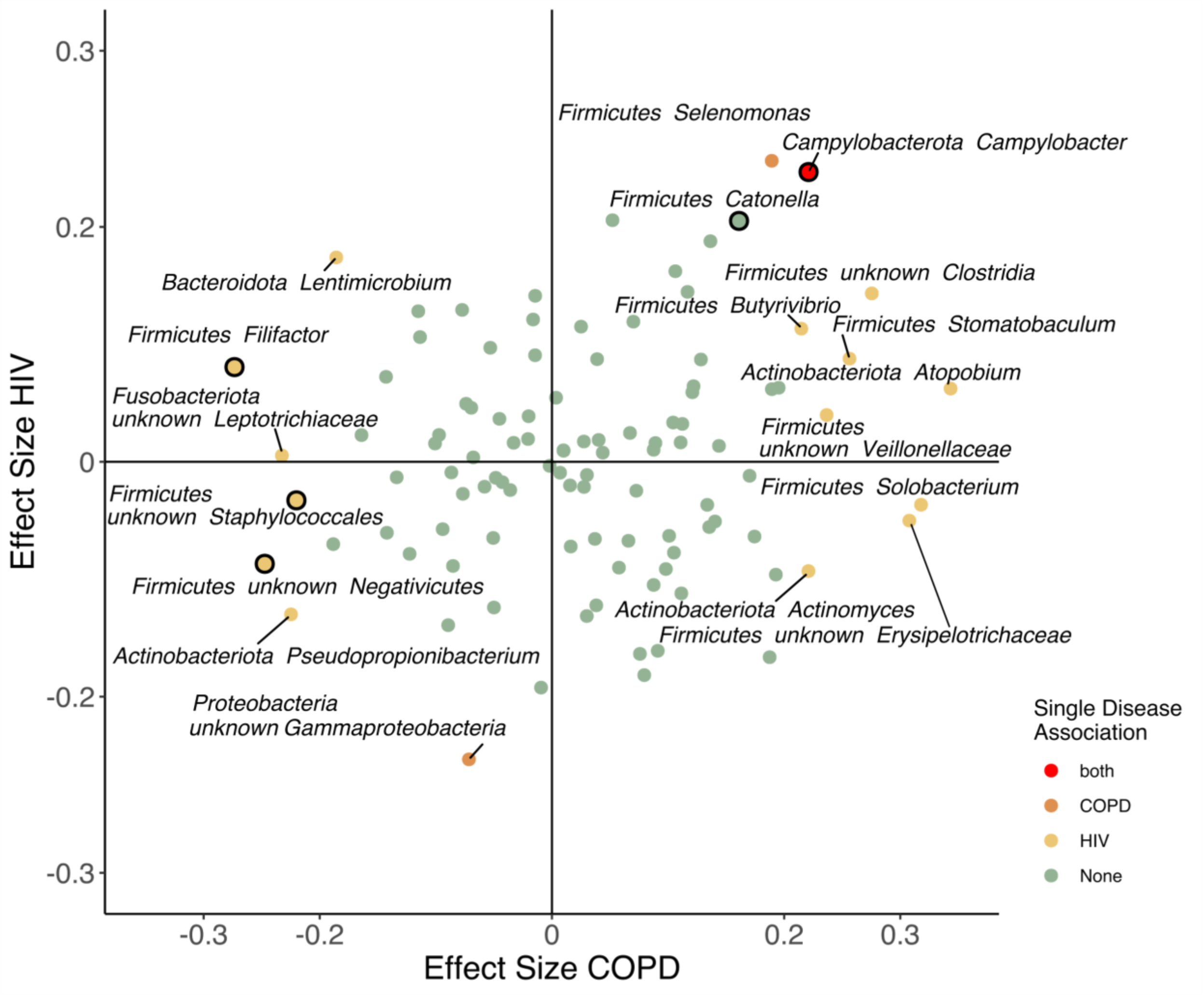
Firmicutes Selenomonas
Campylobacterota Campylobacter
Firmicutes Catonella
Bacteroidota Lentimicrobium
Firmicutes unknown Clostridia
Firmicutes Butyrivibrio
Firmicutes Stomatobaculum
Firmicutes Filifactor
Actinobacteriota Atopobium
Fusobacteriota
unknown Leptotrichiaceae
Firmicutes
unknown Veillonellaceae
Firmicutes
unknown Staphylococcales
Firmicutes Solobacterium
Firmicutes unknown Negativicutes
Actinobacteriota Actinomyces
Firmicutes unknown Erysipelotrichaceae
Actinobacteriota Pseudopropionibacterium
Proteobacteria
unknown Gammaproteobacteria
Effect Size HIV
Effect Size COPD
0.3
0.2
0
-0.2
-0.3
-0.3
-0.2
0
0.2
0.3
Single Disease Association
both
COPD
HIV
None

Figure S3

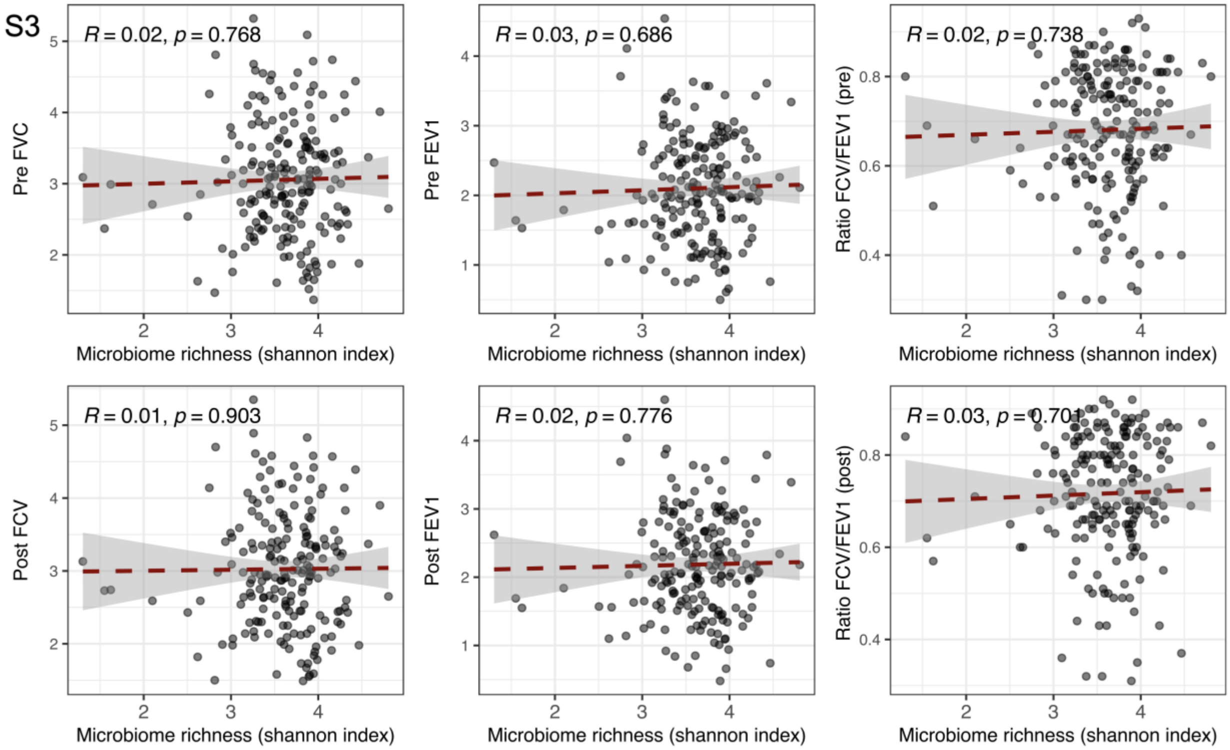
S3
R = 0.02, p = 0.768
Pre FVC
Microbiome richness (shannon index)
R = 0.03, p = 0.686
Pre FEV1
Microbiome richness (shannon index)
R = 0.02, p = 0.738
Ratio FCV/FEV1 (pre)
Microbiome richness (shannon index)
R = 0.01, p = 0.903
Post FCV
Microbiome richness (shannon index)
R = 0.02, p = 0.776
Post FEV1
Microbiome richness (shannon index)
R = 0.03, p = 0.701
Ratio FCV/FEV1 (post)
Microbiome richness (shannon index)

Figure S4

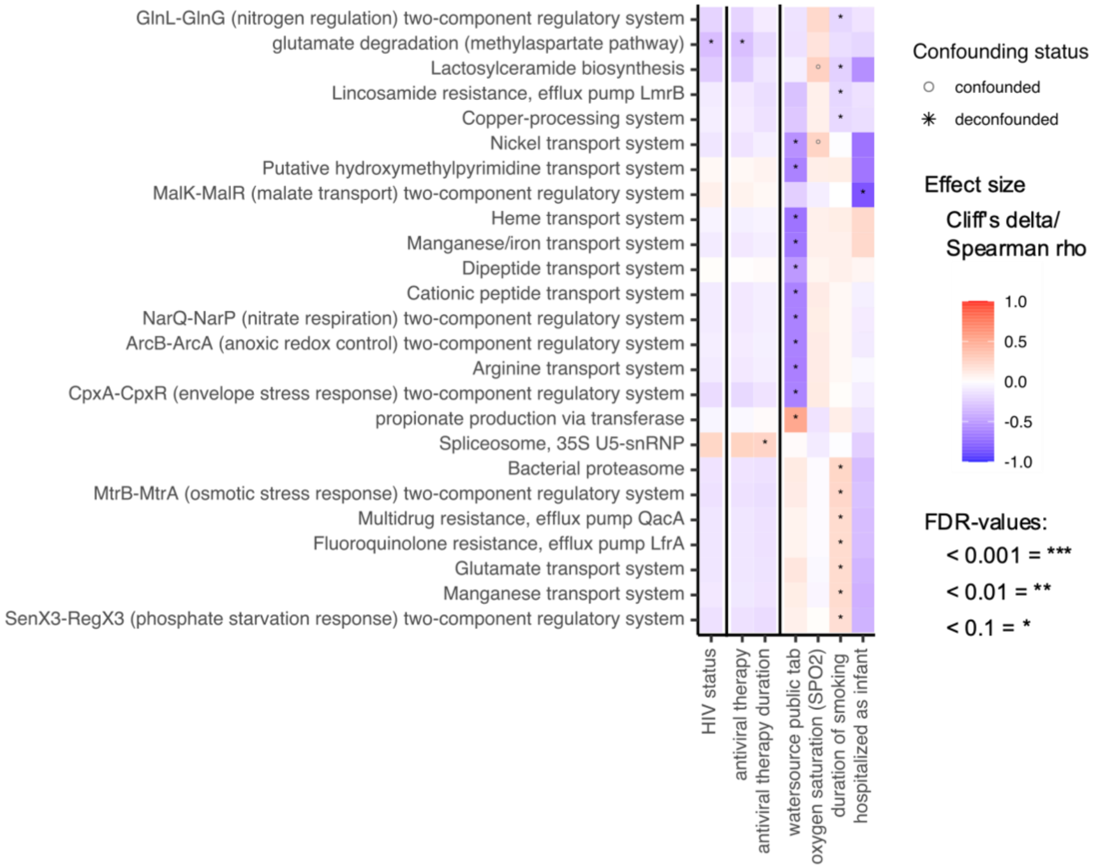

GlnL-GlnG (nitrogen regulation) two-component regulatory system
glutamate degradation (methylaspartate pathway)
Lactosylceramide biosynthesis
Lincosamide resistance, efflux pump LmrB
Copper-processing system
Nickel transport system
Putative hydroxymethylpyrimidine transport system
MalK-MalR (malate transport) two-component regulatory system
Heme transport system
Manganese/iron transport system
Dipeptide transport system
Cationic peptide transport system
NarQ-NarP (nitrate respiration) two-component regulatory system
ArcB-ArcA (anoxic redox control) two-component regulatory system
Arginine transport system
CpxA-CpxR (envelope stress response) two-component regulatory system
propionate production via transferase
Spliceosome, 35S U5-snRNP
Bacterial proteasome
MtrB-MtrA (osmotic stress response) two-component regulatory system
Multidrug resistance, efflux pump QacA
Fluoroquinolone resistance, efflux pump LfrA
Glutamate transport system
Manganese transport system
SenX3-RegX3 (phosphate starvation response) two-component regulatory system
HIV status
antiviral therapy
antiviral therapy duration
watersource public tab
oxygen saturation (SPO2)
duration of smoking
hospitalized as infant
Confounding status
confounded
deconfounded
Effect size
Cliff's delta/
Spearman rho
1.0
0.5
0.0
-0.5
-1.0
FDR-values:
< 0.001 = ***
< 0.01 = **
< 0.1 = *

Figure S5

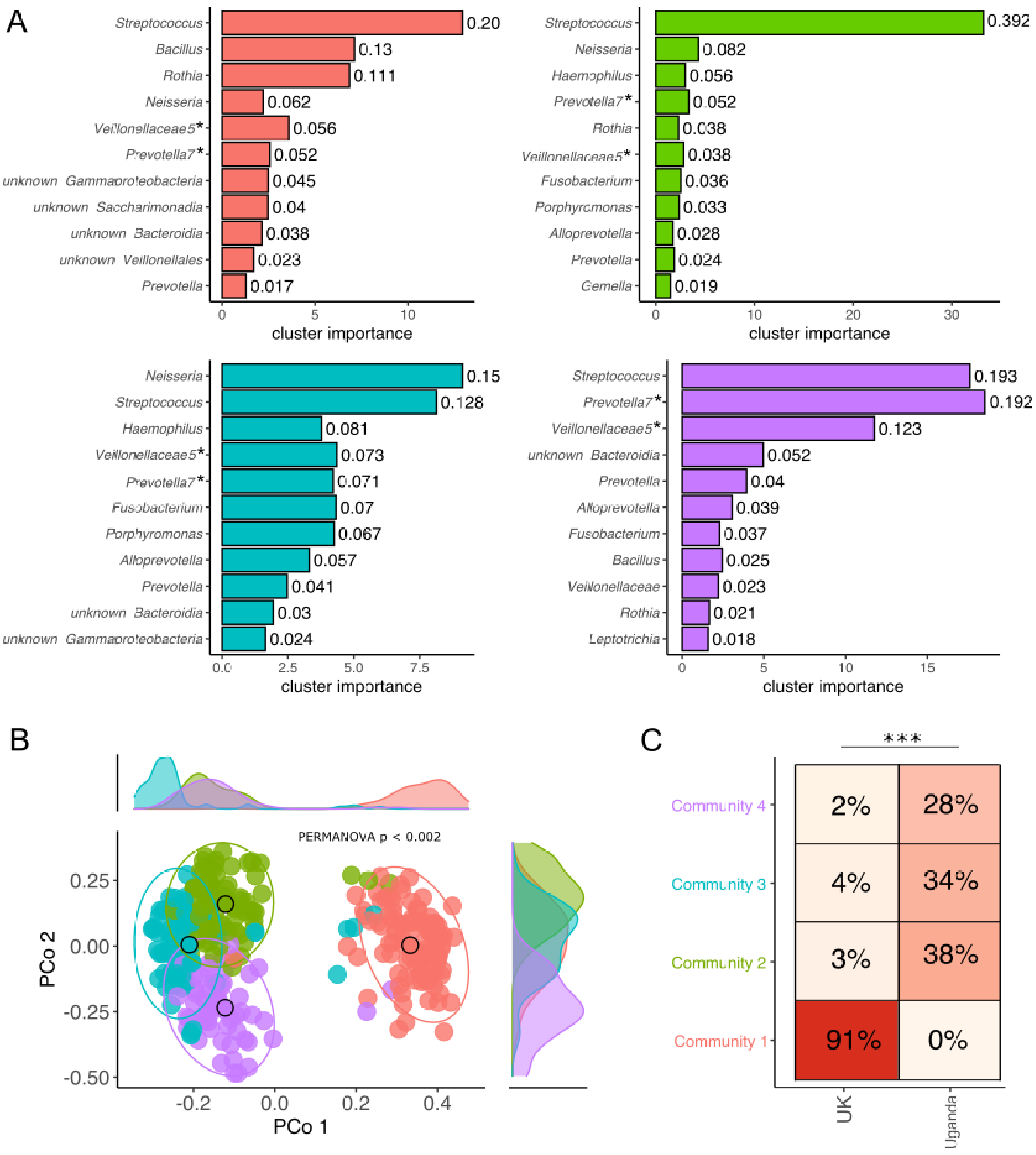
A
Streptococcus 0.20
Bacillus 0.13
Rothia 0.111
Neisseria 0.062
Veillonellaceae5* 0.056
Prevotella7* 0.052
unknown Gammaproteobacteria 0.045
unknown Saccharimonadia 0.04
unknown Bacteroidia 0.038
unknown Veillonellales 0.023
Prevotella 0.017
cluster importance
Streptococcus 0.392
Neisseria 0.082
Haemophilus 0.056
Prevotella7* 0.052
Rothia 0.038
Veillonellaceae5* 0.038
Fusobacterium 0.036
Porphyromonas 0.033
Alloprevotella 0.028
Prevotella 0.024
Gemella 0.019
cluster importance
Neisseria 0.15
Streptococcus 0.128
Haemophilus 0.081
Veillonellaceae5* 0.073
Prevotella7* 0.071
Fusobacterium 0.07
Porphyromonas 0.067
Alloprevotella 0.057
Prevotella 0.041
unknown Bacteroidia 0.03
unknown Gammaproteobacteria 0.024
cluster importance
Streptococcus 0.193
Prevotella7* 0.192
Veillonellaceae5* 0.123
unknown Bacteroidia 0.052
Prevotella 0.04
Alloprevotella 0.039
Fusobacterium 0.037
Bacillus 0.025
Veillonellaceae 0.023
Rothia 0.021
Leptotrichia 0.018
cluster importance
B
PERMANOVA p < 0.002
PCo 1
PCo 2
C
***
Community 4 2% 28%
Community 3 4% 34%
Community 2 3% 38%
Community 1 91% 0%
UK
Uganda

Figure S6

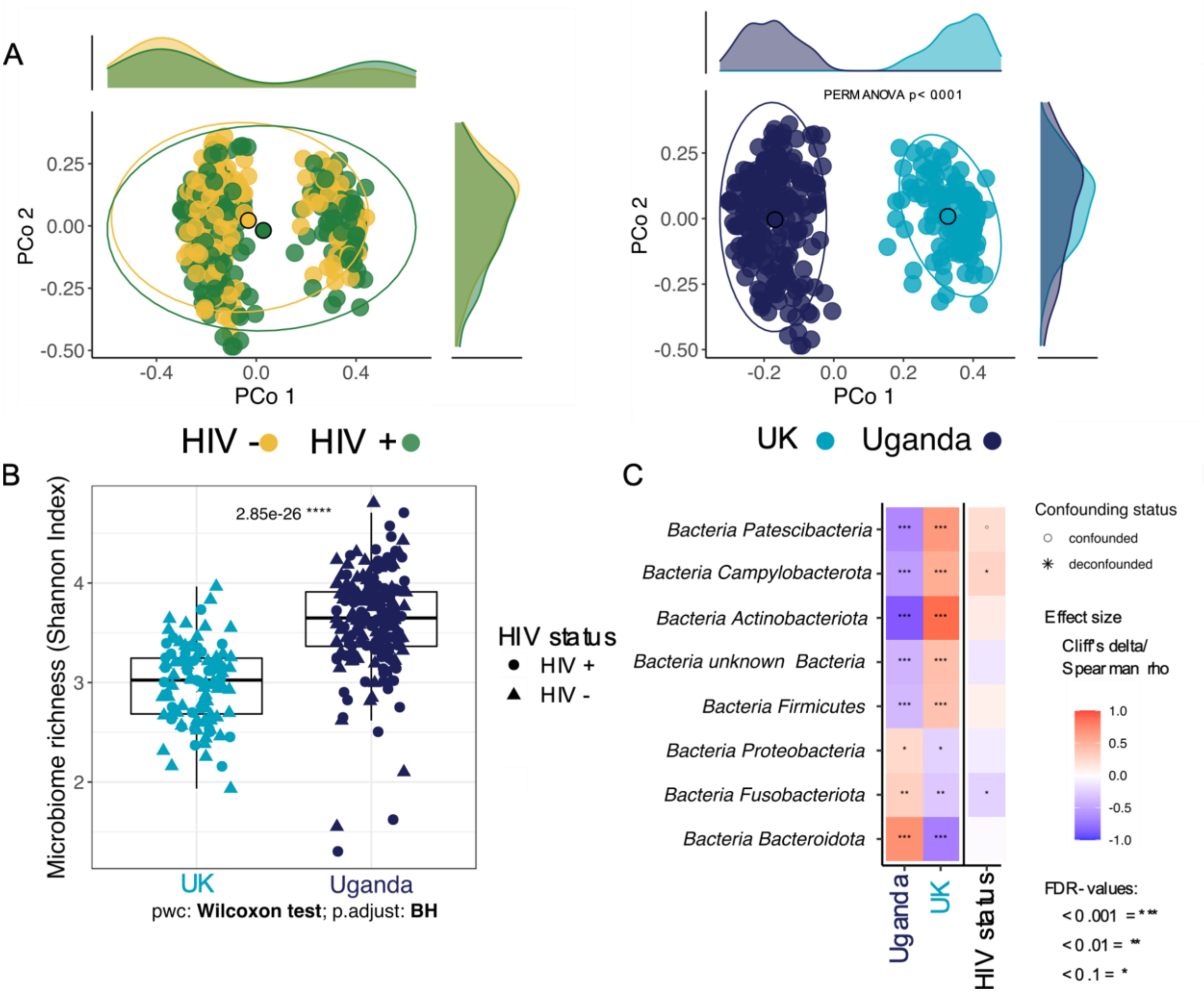
A
PERMANOVA p< 0.001
PCo 2
PCo 1
0.25
0.00
-0.25
-0.50
-0.4
0.0
0.4
-0.2
0.2
HIV - HIV +
UK Uganda
B
2.85e-26 ****
Microbiome richness (Shannon Index)
UK
Uganda
pwc: Wilcoxon test; p.adjust: BH
HIV status
HIV +
HIV -
C
Bacteria Patescibacteria
Bacteria Campylobacterota
Bacteria Actinobacteriota
Bacteria unknown Bacteria
Bacteria Firmicutes
Bacteria Proteobacteria
Bacteria Fusobacteriota
Bacteria Bacteroidota
Uganda
UK
HIV status
Confounding status
confounded
deconfounded
Effect size
Cliff's delta/
Spearman rho
1.0
0.5
0.0
-0.5
-1.0
FDR-values:
< 0.001 = ***
< 0.01 = **
< 0.1 = *

Figure S7

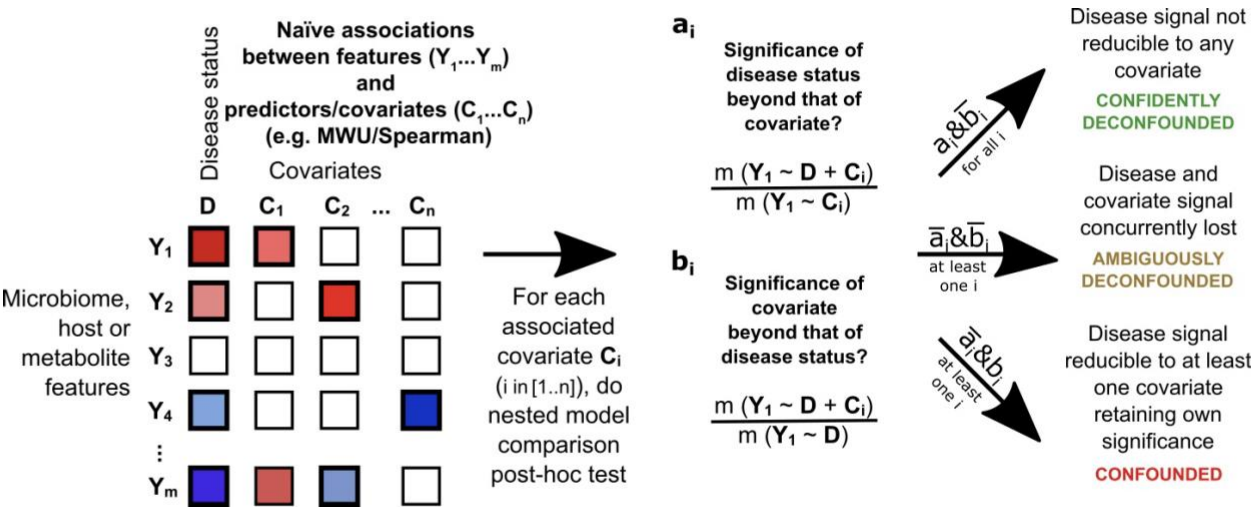
Naïve associations between features (Y_1...Y_m) and predictors/covariates (C_1...C_n) (e.g. MWU/Spearman)
Disease status
Covariates
D
C_1
C_2
...
C_n
Y_1
Y_2
Y_3
Y_4
Y_m
Microbiome, host or metabolite features
For each associated covariate C_i (i in [1..n]), do nested model comparison post-hoc test
a_i
Significance of disease status beyond that of covariate?
$\frac{m(Y_1 \sim D + C_i)}{m(Y_1 \sim C_i)}$
b_i
Significance of covariate beyond that of disease status?
$\frac{m(Y_1 \sim D + C_i)}{m(Y_1 \sim D)}$
$a_i \& \bar{b}_i$
for all i
$\bar{a}_i \& \bar{b}_i$
at least one i
$\bar{a}_i \& b_i$
at least one i
Disease signal not reducible to any covariate
CONFIDENTLY DECONFOUNDED
Disease and covariate signal concurrently lost
AMBIGUOUSLY DECONFOUNDED
Disease signal reducible to at least one covariate retaining own significance
CONFOUNDED

Table ST1: Raw reads before and after filtering

Sample ID	Before filtering	After filtering
A01.LMB001	35400	34774
A02.LMB009	37409	37319
A03.LMB017	18097	18091
A04.LMB025	43895	43749
A05.LMB033	38365	38243
A06.LMB041	29758	29513
A07.LMB049	27153	27152
A08.LMB058	52176	52160
A09.LMB066	32454	32448
A10.LMB074	42375	42330
A11.LMB082	24017	24016
A12.LMB090	49862	49848
B01.LMB002	61307	61282
B02.LMB010	28982	26124
B03.LMB018	28755	28555
B04.LMB026	44481	44209
B05.LMB034	67173	67101
B06.LMB042	32485	32429
B07.LMB051	44024	43881
B08.LMB059	56991	56706
B09.LMB067	46670	46374
B10.LMB075	26453	26444
B11.LMB083	31220	31164
B12.LMB091	46987	46611
C01.LMB003	40372	40290
C02.LMB011	43804	43681
C03.LMB019	33503	32941
C04.LMB027	48960	48894
C05.LMB035	51775	50673
C06.LMB043	47034	47020
C07.LMB052	45270	44600
C08.LMB060	65230	65218
C09.LMB068	48515	47676
C10.LMB076	50361	50358
C11.LMB084	52291	51816

C12.LMB093	43854	43828
D01.LMB004	39406	39008
D02.LMB012	59800	59686
D03.LMB020	27460	27270
D04.LMB028	31676	28391
D05.LMB036	23370	12658
D06.LMB044	54350	54158
D07.LMB053	24313	24305
D08.LMB061	10842	9962
D09.LMB069	55809	55735
D10.LMB077	63567	63479
D11.LMB085	33450	33422
D12.LMB094	60469	60309
E01.LMB005	51110	51107
E02.LMB013	44803	44661
E03.LMB021	17966	17908
E04.LMB029	66028	65980
E05.LMB037	38819	38552
E06.LMB045	45076	44859
E07.LMB054	26461	26432
E08.LMB062	69308	69296
E09.LMB070	49199	49190
E10.LMB078	36744	36742
E11.LMB086	30898	30896
E12.LMB095	78966	78962
F01.LMB006	12289	10976
F02.LMB014	27058	26096
F03.LMB022	46149	46044
F04.LMB030	14400	13496
F05.LMB038	52014	51867
F06.LMB046	59217	59070
F07.LMB055	42737	42481
F08.LMB063	22469	22467
F09.LMB071	24643	24561
F10.LMB079	46196	46173
F11.LMB087	53815	53803
F12.LMB096	26356	26349
G01.LMB007	54517	53635

G02.LMB015	49781	49285
G03.LMB023	29600	29560
G04.LMB031	27129	26944
G05.LMB039	104695	104606
G06.LMB047	51554	51529
G07.LMB056	38273	38205
G08.LMB064	41093	41074
G09.LMB072	61641	61564
G10.LMB080	43307	43296
G11.LMB088	44326	44031
G12.LMB097	47791	47781
H01.LMB008	84413	82843
H02.LMB016	37509	23868
H03.LMB024	48852	48751
H04.LMB032	53700	52960
H05.LMB040	53046	48619
H06.LMB048	60991	60435
H07.LMB057	55598	55322
H08.LMB065	41260	40320
H09.LMB073	58859	58740
H10.LMB081	61592	61559
H11.LMB089	59507	59476
H12.LMB098	60678	60434
A01.LMB099	32351	32163
A02.LMB108	36519	36299
A03.LMB116	25728	25638
A04.LMB125	42343	42316
A05.LMB133	24422	24312
A06.LMB141	25512	25365
A07.LMB149	13992	13989
A08.LMB157	41324	41320
A09.LMB165	29596	28842
A10.LMB173	39723	39641
A11.LMB182	22042	21969
A12.LMB190	52193	52180
B01.LMB101	51492	51333
B02.LMB109	34632	34479
B03.LMB117	26379	25249

B04.LMB126	23335	20966
B05.LMB134	34360	34351
B06.LMB142	31585	31572
B07.LMB150	25665	25665
B08.LMB158	33265	32859
B09.LMB166	58077	57954
B10.LMB174	37062	37046
B11.LMB183	35073	34714
B12.LMB191	41090	39793
C01.LMB102	57845	57646
C02.LMB110	60645	60640
C03.LMB118	37097	37079
C04.LMB127	62167	62145
C05.LMB135	30886	30079
C06.LMB143	33676	26715
C07.LMB151	30846	30822
C08.LMB159	38957	38949
C09.LMB167	44153	42992
C10.LMB175	53286	53272
C11.LMB184	24964	21471
C12.LMB192	63384	62624
D01.LMB103	62885	62823
D02.LMB111	70014	69638
D03.LMB119	32275	32256
D04.LMB128	57209	56658
D05.LMB136	33478	31020
D06.LMB144	40475	38449
D07.LMB152	20973	20174
D08.LMB160	45384	45232
D09.LMB168	36183	36109
D10.LMB176	50768	47285
D11.LMB185	27164	24249
D12.LMB193	71177	70929
E01.LMB104	58777	58771
E02.LMB112	47103	47008
E03.LMB120	32074	32066
E04.LMB129	97622	97461
E05.LMB137	35570	35563

E06.LMB145	37035	37030
E07.LMB153	26546	26538
E08.LMB161	56904	56896
E09.LMB169	46331	46301
E10.LMB177	70080	70052
E11.LMB186	22251	22002
E12.LMB194	68056	67974
F01.LMB105	38223	38117
F02.LMB113	48342	47914
F03.LMB121	27782	27047
F04.LMB130	31214	31147
F05.LMB138	31557	31424
F06.LMB146	52015	52003
F07.LMB154	31216	31069
F08.LMB162	9431	9252
F09.LMB170	37962	37938
F10.LMB178	49031	48823
F11.LMB187	33307	32337
F12.LMB195	27385	27372
G01.LMB106	52555	52332
G02.LMB114	49897	49872
G03.LMB123	31262	30686
G04.LMB131	52950	52494
G05.LMB139	46345	45142
G06.LMB147	41396	41295
G07.LMB155	26585	26557
G08.LMB163	23893	22585
G09.LMB171	70533	69915
G10.LMB179	56865	56752
G11.LMB188	37005	36826
G12.LMB196	51035	50934
H01.LMB107	84697	70631
H02.LMB115	59260	58535
H03.LMB124	50915	50678
H04.LMB132	71203	70009
H05.LMB140	55810	55783
H06.LMB148	45982	45688
H07.LMB156	50488	50467

H08.LMB164	35210	34773
H09.LMB172	67594	67554
H10.LMB181	65422	65114
H11.LMB189	70219	69609
H12.LMB197	74820	74747
A01.LMB198	34077	33665
B01.LMB199	31121	26550
C01.LMB200	34969	31955
D01.LMB201	40233	39075
E01.LMB202	49522	49266
F01.LMB203	26708	26149
G01.LMB204	44760	41730
H01.LMB205	28533	22355
